# Mortality and neurological outcomes in extremely and very preterm infants born to mothers with hypertensive disorders of pregnancy

**DOI:** 10.1038/s41598-021-81292-7

**Published:** 2021-01-18

**Authors:** Noriyuki Nakamura, Takafumi Ushida, Masahiro Nakatochi, Yumiko Kobayashi, Yoshinori Moriyama, Kenji Imai, Tomoko Nakano-Kobayashi, Masahiro Hayakawa, Hiroaki Kajiyama, Fumitaka Kikkawa, Tomomi Kotani, for the Neonatal Research Network of Japan

**Affiliations:** 1 Department of Obstetrics and Gynecology, Nagoya University Graduate School of Medicine, 65 Tsurumai-cho, Showa-ku, Nagoya, 466-8550 Japan; 2 Division of Public Health Informatics, Department of Integrative Health Science, Nagoya University Graduate School of Medicine, Nagoya, Japan; 3 Data Science Division, Data Coordinating Center, Department of Advanced Medicine, Nagoya University Hospital, Nagoya, Japan; 4 Department of Obstetrics and Gynecology, Fujita Health University School of Medicine, Toyoake, Japan; 5 Division of Neonatology, Center for Maternal-Neonatal Care, Nagoya University Hospital, Nagoya, Japan; 6 Division of Perinatology, Center for Maternal-Neonatal Care, Nagoya University Hospital, Nagoya, Japan

**Keywords:** Neonatal brain damage, Neonatology, Preterm birth

## Abstract

To evaluate the impact of maternal hypertensive disorders of pregnancy (HDP) on mortality and neurological outcomes in extremely and very preterm infants using a nationwide neonatal database in Japan. This population-based retrospective study was based on an analysis of data collected by the Neonatal Research Network of Japan from 2003 to 2015 of neonates weighing 1,500 g or less at birth, between 22 and 31 weeks’ gestation. A total of 21,659 infants were randomly divided into two groups, HDP (n = 4,584) and non-HDP (n = 4,584), at a ratio of 1:1 after stratification by four factors including maternal age, parity, weeks of gestation, and year of delivery. Short-term (neonatal period) and medium-term (3 years of age) mortality and neurological outcomes were compared between the two groups by logistic regression analyses. In univariate analysis, HDP was associated with an increased risk for in-hospital death (crude odds ratio [OR], 1.31; 95% confidence interval, 1.04–1.63) and a decreased risk for severe intraventricular haemorrhage (0.68; 0.53–0.87) and periventricular leukomalacia (0.60; 0.48–0.77). In multivariate analysis, HDP was significantly associated with a lower risk for in-hospital death (adjusted OR, 0.61; 0.47–0.80), severe intraventricular haemorrhage (0.47; 0.35–0.63), periventricular leukomalacia (0.59; 0.45–0.78), neonatal seizures (0.40; 0.28–0.57) and cerebral palsy (0.70; 0.52–0.95) at 3 years after adjustment for covariates including birth weight. These results were consistent with those of additional analyses, which excluded cases with histological chorioamnionitis and which divided the infants into two subgroups (22–27 gestational weeks and 28–31 gestational weeks). Maternal HDP was associated with an increased risk for in-hospital death without adjusting for covariates, but it was also associated with a lower risk for mortality and adverse neurological outcomes in extremely and very preterm infants if all covariates except HDP were identical.

## Introduction

Hypertensive disorders of pregnancy (HDP) affects 5–13% of all pregnancies^1,2^ and is a significant cause of mortality and morbidity for both the affected mothers and their infants^3^. HDP is characterised by hypertension during pregnancy and includes preeclampsia, which is accompanied by endothelial dysfunction and defined as a new onset of hypertension with either proteinuria or end-organ dysfunction after 20 weeks of gestation^4,5^. Approximately 20–50% of infants born to mothers with HDP develop fetal growth restriction (FGR) because of placental dysfunction^6,7^, and these affected infants are susceptible to lifelong adverse consequences and a poor quality of life^8,9^. 

Increasing evidence suggests that intrauterine exposure to maternal HDP has a negative impact on preterm infants’ neurological outcomes including cerebral palsy (CP), low developmental quotient (DQ) and cognitive dysfunction^10,11^. Additionally, recent meta-analyses have found that infants exposed to maternal HDP have an increased risk for autism spectrum disorders or attention deficit hyperactivity disorder^12,13^. Conversely, several reports have found a lower mortality rate and a lower incidence of severe brain injuries such as intraventricular haemorrhage (IVH) or periventricular leukomalacia (PVL) among very preterm infants born to mothers with HDP^14–16^. Currently, the impact of maternal HDP on neurological outcomes in infants, especially very preterm infants, is still controversial. In addition, little evidence is available regarding the association between maternal HDP and early childhood neurological outcomes in very preterm infants.

The aim of this study was to evaluate the impact of maternal HDP on the short-term (neonatal period) and medium-term (3 years of age) mortality and neurological outcomes of extremely and very preterm infants using a nationwide neonatal database.

## Methods

### Study population

This was a retrospective cohort study based on the database of the Neonatal Research Network of Japan (NRNJ) from January 2003 to December 2015. Data on neonates born before 32 weeks’ gestation and weighing ≤ 1,500 g were prospectively collected from perinatal centres participating in the NRNJ since 2003. The number of Level II and III neonatal intensive care units (NICUs) participating in the NRNJ has been increasing in recent years. By 2015, there were more than 150 units with approximately 3,000–4,000 neonates being registered in the database every year^17^. Information on maternal characteristics, neonatal outcomes and postnatal management were collected by manual research, anonymised, and then registered in the NRNJ database. Written informed consent was obtained from all parents of the infants at each participating institution. This study was approved by the institutional ethics board of Nagoya University (approval number: 2018–0026, approval date: 9th May, 2018) and the Japan Neonatal Network Executive Committee. All research was performed in accordance with relevant guidelines and regulations.

**Figure 1:**
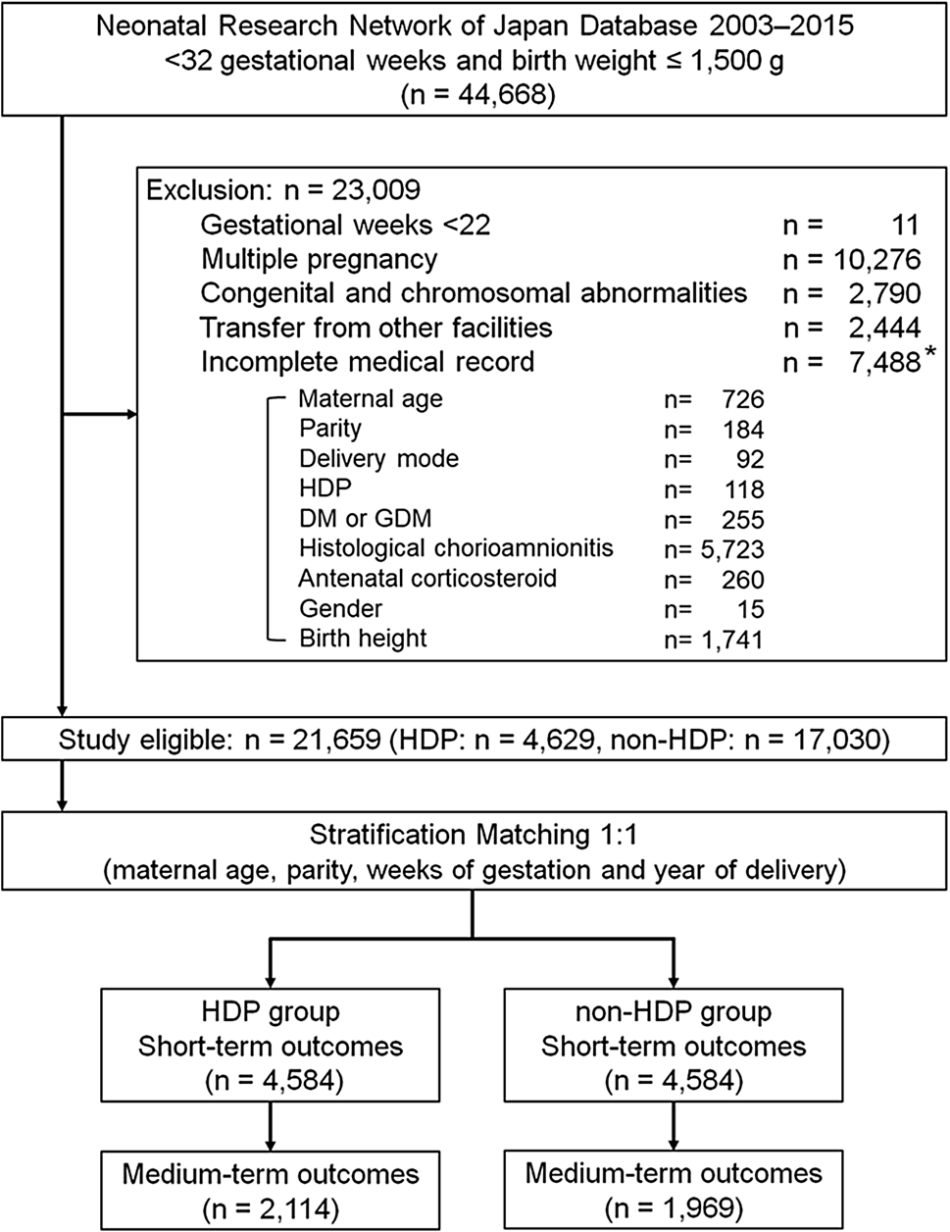
Study enrolment. Data on 44,668 neonates born at NICUs participating in the Neonatal Research Network of Japan from January 2003 to December 2015 were available. Each of 4,584 neonates in the HDP and non-HDP groups was selected from eligible infants (HDP, n = 4,629; non-HDP, n = 17,030) after stratification matching using four factors, including maternal age, parity, weeks of gestation, and year of delivery. *Items not mutually exclusive. GDM, gestational diabetes mellitus; DM, diabetes mellitus; HDP, hypertensive disorders of pregnancy.

Singleton neonates weighing ≤ 1,500 g and born between 22 and 31 weeks’ gestation were included in this study (Fig. 1). Multiple pregnancies, infants with congenital and chromosomal abnormalities, infants transferred from other facilities, or infants with incomplete medical records were excluded. Supplementary Table 1 lists the baseline maternal and neonatal characteristics with and without complete data (n = 21,659 and n = 7,488, respectively). After exclusion of 22,998 infants, a total of 21,659 infants remained (HDP, n = 4,629; non-HDP, n = 17,030). They were randomly divided into two groups (HDP and non-HDP groups) at a ratio of 1:1 after stratification by four factors: maternal age at delivery (10’s, 20’s, 30’s and 40’s and over), parity (primiparous and multiparous), weeks of gestation at delivery (22–23, 24–25, 26–27, 28–29 and 30–31 weeks) and year of delivery (between 2003–2008 and between 2009–2015). After 1:1 stratification matching, 4,584 infants in each group were selected for further analyses (Fig. 1).

### Definition and outcomes

HDP was defined as hypertension (systolic blood pressure ≥ 140 mmHg and/or diastolic blood pressure ≥ 90 mmHg) during pregnancy^18^. The subtype and severity of HDP were not recorded in the NRNJ database. IVH was diagnosed by the intracranial ultrasound. The grade of IVH was defined by the Papile classification^19^, and severe IVH was defined as grade III or IV. PVL was diagnosed if cystic PVL was detected by intracranial ultrasound or brain magnetic resonance imaging. Neonatal seizures were diagnosed on the basis of clinical symptoms or amplitude-integrated electroencephalograms. Medium-term neurological outcomes such as CP and DQ were evaluated at approximately 36 months of chronological age. CP was defined as permanent disorders of development of movement and posture caused by non-progressive disturbances of the fetal or infant brain^20^. However, the subtype and severity of CP were not documented in the NRNJ database. The Kyoto Scale of Psychologic Development (KSPD) was used for the evaluation of DQ. The KSPD comprises three categories including Postural-Motor (P-M), Cognitive-Adaptive (C-A) and Language-Social (L-S) areas. We calculated DQ by averaging the scores of the three categories and adjusting according to developmental or chronological age. In this study, DQ at 3 years of age was adjusted according to chronological age. In general, DQ of < 70 indicates neurodevelopmental delay. The KSPD is a neurodevelopmental assessment tool for use in early childhood in Japan, and it is well correlated with the Bayley III Cognitive and Language scales as previously reported^21,22^. Short-term outcomes (severe IVH, PVL, neonatal seizures and in-hospital death) were evaluated at NICU discharge, and medium-term outcomes (CP, DQ of < 70 and death from birth to 3 years of age) were evaluated at approximately 36 months of age.

### Statistical analysis

Continuous and categorical variables were compared using Student’s *t* test or chi-square test, respectively. The short- and medium-term neurological outcomes were compared between the HDP and non-HDP groups. Crude odds ratios (ORs) and 95% confidence intervals (CIs) were evaluated by univariate logistic regression models. We then evaluated the adjusted ORs using three types of multivariate logistic regression models after the adjustment for covariates including diabetes mellitus (DM) or gestational diabetes mellitus (GDM), histological chorioamnionitis (CAM), administration of antenatal corticosteroids, mode of delivery, infant sex, and birth weight (model 1, including birth weight; model 2, excluding birth weight and; model 3, including small for gestational age [SGA] instead of birth weight). We then evaluated the incidence of mortality and adverse neurological outcomes stratified by weeks of gestation at birth without adjustment of covariates. In addition, two subgroup analyses were performed to eliminate the negative effect of histological CAM on neonatal outcomes and to confirm whether our results were consistent within the different ranges of gestational age at birth. First, we analysed outcomes after excluding cases with histological CAM from both groups. Second, we evaluated outcomes after infants in both groups were divided into two subgroups (22–27 weeks and 28–31 weeks of gestation at birth). Statistical significance was defined as a *p*-value < 0.05. All statistical analyses were performed with SAS version 9.4 (SAS Institute Inc., Cary, NC, USA).

## Results

During the study period, 44,668 infants were registered in the NRNJ database. After 22,998 infants were excluded, the remaining 21,659 infants were randomly divided into two groups after 1:1 stratification matching (HDP, n = 4,584; non-HDP, n = 4,584; Fig. 1). The baseline characteristics of the two groups are noted in Table 1. Characteristics such as maternal age, parity, weeks of gestation and year of delivery were completely matched between the two groups (*p* = 1.0, Table 1). Before stratification matching, the baseline characteristics were significantly different between the two groups over all variables (Supplementary Table 2). Following stratification matching, mothers in the HDP group were more likely to have delivered by caesarean section and experience GDM or DM. Mothers in the non-HDP group had a significantly increased incidence of premature rupture of the membranes and histological CAM, and they were more likely to have received antenatal corticosteroid treatment. Neonates in the HDP group were more likely to be female with lower birth weight and height and have a significantly increased rate of SGA (Table 1). Supplementary Fig. 1 presents a graph showing gestational age (day) plotted against birth weight (g) with fitted curves for the two groups (red represents HDP [n = 4,584]; blue represents non-HDP [n = 4,584]) to depict the distribution. We found that the birth weight of neonates in the HDP group was significantly lower than that of neonates in the non-HDP group after stratification matching.

**Table 1:** Baseline characteristics after 1:1 stratification matching in the HDP and non-HDP groups.

	HDP	non-HDP	
Variables	(n = 4,584)	(n = 4,584)	*p-*value
Maternal characteristics			
Maternal age (years)	33.8 ± 5.0	33.0 ± 5.1	
**Category of age**			1.0
10’s	22 (0.5%)	22 (0.5%)	
20’s	883 (19.3%)	883 (19.3%)	
30’s	3,152 (68.8%)	3,152 (68.8%)	
40’s and over	527 (11.5%)	527 (11.5%)	
Primiparous	2,632 (57.4%)	2,632 (57.4%)	1.0
Gestational age (weeks)	28.8 ± 2.2	28.7 ± 2.1	
**Category of gestational weeks**			1.0
22–23 weeks	84 (1.8%)	84 (1.8%)	
24–27 weeks	1,402 (30.6%)	1,402 (30.6%)	
28–31 weeks	3,098 (67.6%)	3,098 (67.6%)	
Caesarean section	4,406 (96.1%)	3,332 (72.7%)	< 0.01
DM or GDM	190 (4.1%)	133 (2.9%)	< 0.01
Histological chorioamnionitis	643 (14.0%)	2,043 (44.6%)	< 0.01
Antenatal corticosteroid	2,460 (53.7%)	2,747 (59.9%)	< 0.01
PROM	188 (4.1%)	1,997 (43.6%)	< 0.01
**Year of delivery**			1.0
2003–2008	1,191 (26.0%)	1,191 (26.0%)	
2009–2015	3,393 (74.0%)	3,393 (74.0%)	
**Neonatal characteristics**			
Male	2,124 (46.3%)	2,467 (53.8%)	< 0.01
Birth weight (g)	913 ± 291	1,090 ± 280	< 0.01
Height (cm)	34.0 ± 4.0	35.9 ± 3.6	< 0.01
SGA	2,477 (54.0%)	772 (16.8%)	< 0.01

Data are given as mean ± standard deviation for continuous variables and n (%) for categorical variables. HDP, hypertensive disorders of pregnancy; DM, diabetes mellitus; GDM, gestational diabetes mellitus; PROM, premature rupture of membranes; SGA, small for gestational age.

**Table 2:** Neurological outcomes in the HDP and non-HDP groups in univariate and multivariate analyses.

Variables	HDP	Non-HDP	Crude OR (95% CI)	Adjusted OR (95% CI)		
				Model 1	Model 2	Model 3
**Short-term outcomes**	(n = 4,584)	(n = 4,584)				
IVH (grade III or IV)	108/4.565 (2.4%)	157/4,565 (3.4%)	**0.68 (0.53–0.87)**	**0.47 (0.35–0.63)**	0.76 (0.58–1.00)	0.82 (0.61–1.09)
PVL	111/4,567 (2.4%)	181/4,568 (4.0%)	**0.60 (0.48–0.77)**	**0.59 (0.45–0.78)**	**0.63 (0.48–0.81)**	**0.69 (0.52–0.90)**
Neonatal seizures	63/4,577 (1.4%)	98/4,579 (2.1%)	**0.64 (0.46–0.88)**	**0.40 (0.28–0.57)**	**0.59 (0.42–0.84)**	**0.61 (0.42–0.88)**
In-hospital death	181/4,583 (3.9%)	140/4,582 (3.1%)	**1.31 (1.04–1.63)**	**0.61 (0.47–0.80)**	**1.34 (1.04–1.72)**	1.11 (0.85–1.44)
**Medium-term outcomes**	(n = 2,114)	(n = 1,969)				
Cerebral palsy	112/1,845 (6.1%)	120/1,737 (6.9%)	0.87 (0.67–1.14)	**0.70 (0.52–0.95)**	0.89 (0.67–1.19)	0.92 (0.68–1.25)
DQ of < 70	216/1,344 (16.1%)	167/1,264 (13.2%)	**1.26 (1.01–1.57)**	0.93 (0.72–1.20)	**1.34 (1.05–1.71)**	1.18 (0.91–1.52)
Total death by 3 years of age	192/2,114 (9.1%)	159/1,969(8.1%)	1.14 (0.91–1.42)	**0.59 (0.45–0.78)**	1.21 (0.95–1.54)	1.02 (0.79–1.31)

Data are given as n (%). Adjusted ORs were evaluated by multivariate analyses (models 1, 2, and 3). Bold text indicates a significant association.

Model 1: diabetes mellitus or gestational diabetes mellitus, histological chorioamnionitis, administration of antenatal corticosteroid, mode of delivery, gender, and birth weight were included in the logistic regression model.

Model 2: diabetes mellitus or gestational diabetes mellitus, histological chorioamnionitis, administration of antenatal corticosteroid, mode of delivery, and gender were included in the logistic regression model.

Model 3: diabetes mellitus or gestational diabetes mellitus, histological chorioamnionitis, administration of antenatal corticosteroid, mode of delivery, gender, and SGA were included in the logistic regression model.

HDP, hypertensive disorders of pregnancy; IVH, intraventricular haemorrhage; PVL, periventricular leukomalacia; DQ, developmental quotient; OR, odds ratio; CI, confidence interval; SGA, small for gestational age.

Table 2 notes mortality and neurological outcomes in the HDP and non-HDP groups by univariate and multivariate analyses. The ORs of IVH (grade III or IV) (crude OR 0.68; 95%CI 0.53–0.87 and adjusted OR 0.47; 0.35–0.63), PVL (crude OR 0.60; 0.48–0.77 and adjusted OR 0.59; 0.45–0.78) and neonatal seizures (crude OR 0.64; 0.46–0.88 and adjusted OR 0.40; 0.28–0.57) were significantly lower in the HDP group compared with the non-HDP group in both univariate and multivariate analyses (model 1; including birth weight). The HDP group had a higher rate of in-hospital death (crude OR 1.31; 1.04–1.63) and DQ of < 70 (crude OR 1.26; 1.01–1.57) on univariate analysis. However, the adjusted ORs of in-hospital death and total death by 3 years of age in the HDP group (adjusted OR 0.61; 0.47–0.80 and 0.59; 0.45–0.78, respectively) were significantly lower in the HDP group after adjustment for several covariates including birth weight (model 1). The risk of CP at 3 years of age (adjusted OR 0.70; 0.52–0.95) was also significantly lower in the HDP group upon multivariate analysis (model 1). However, the adjusted ORs of several outcomes differed considerably based on the covariates included in the specific models (model 2 [excluding birth weight] and model 3 [including SGA instead of birth weight]).

To eliminate the effect of CAM, which is also significantly associated with poor neurodevelopmental outcomes, we performed a subgroup analysis excluding infants born to mothers with histological CAM (Supplementary Table 3). Even after exclusion of these infants from both groups, the neurological outcomes in the HDP group were better than those in the non-HDP group upon multivariate analysis.

**Figure 2:**
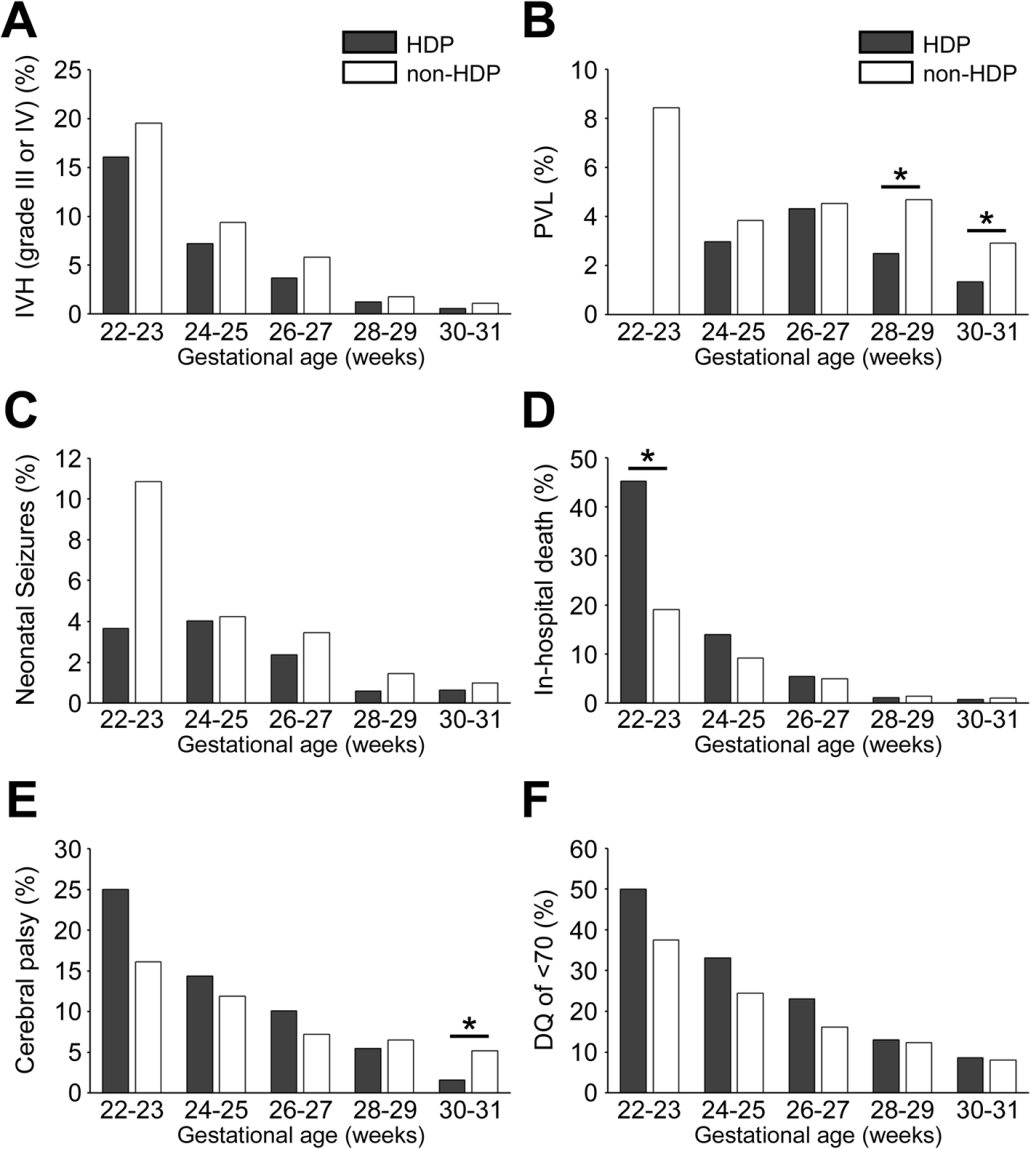
Mortality and severe neurological outcomes by gestational age at birth in the HDP and non-HDP groups. The black and white bars show the rates of neurological outcomes in the HDP and non-HDP groups, respectively. HDP, hypertensive disorders of pregnancy; IVH, intraventricular haemorrhage; PVL, periventricular leukomalacia; CP, cerebral palsy; DQ, developmental quotient. *p < 0.01 for HDP versus non-HDP.

Figure 2 shows the infants’ neurological outcomes stratified by gestational age without adjustment for any factors. Although the HDP group had a higher rate of in-hospital death between 22 and 25 weeks of gestation, the incidence of IVH (grade III or IV), PVL and neonatal seizures tended to be lower in the HDP group than in the non-HDP group. The incidence of CP and DQ of < 70 at 3 years of age tend to be higher in the HDP group than in the non-HDP group prior to 28 weeks of gestation.

After stratification by gestational age but without adjustment for any factors, the HDP group < 28 weeks of gestation had worse outcomes, including in-hospital death, CP and DQ of < 70 at 3 years of age. To determine whether these results were dependent on gestational age, we performed additional multivariate analyses in which the HDP and non-HDP groups were each divided in two (22–27 weeks and 28–31 weeks of gestation). The crude OR of in-hospital death (OR 1.52; 1.17–1.97) was higher in the HDP group < 28 weeks’ gestation in univariate analysis; however, the adjusted OR (adjusted OR 0.60; 0.43–0.84 was lower in the HDP group upon multivariate analysis (Supplementary Table 4). Among infants born at 28–31 weeks of gestation, the adjusted ORs of all short-term outcomes and CP at 3 years of age were significantly lower in the HDP group upon multivariate analysis (Supplementary Table 5).

## Discussion

The aim of this study was to evaluate the impact of HDP on short- and medium-term mortality and neurological outcomes in extremely and very preterm infants. Among infants born at less than 32 weeks’ gestation and weighing ≤ 1,500 g, those born to mothers with HDP are associated with an increased risk for in-hospital death and DQ of < 70 at 3 years of age without adjusting for any covariates; however, they are associated with a lower risk for short- and medium-term mortality, IVH (grade III or IV), PVL and CP compared with those without HDP after adjustment for covariates including birth weight. This result was supported by additional subgroup analyses to eliminate the effect of CAM on outcomes and to confirm whether these results were consistent with two different ranges of gestational age (22–27 weeks and 28–31 weeks of gestation). Our study suggested that the mortality and neurological outcomes for infants born to mothers with HDP were better than those for infants without HDP among extremely and very preterm infants if the background of the infants is exactly the same except for the presence of HDP.

The adjusted ORs of severe brain injury in the HDP group were approximately half the ORs in the non-HDP group based on multivariate analyses. Our results were similar to prior studies, which showed a reduced risk of severe brain injury in preterm infants born to mothers with HDP^14–16^. However, in the unadjusted analysis, stratified by gestational age, the incidence of in-hospital death, CP and DQ of < 70 in the HDP group was higher than that of the non-HDP group before 28 weeks’ gestation. This was also consistent with prior reports^6,11,16^.

These conflicting results can be accounted for in part by which covariates were included in the logistic regression models. In particular, whether birth weight is included or not in the models may have significant impact on the adjusted ORs of several outcomes in this study. A recent study demonstrated that of six factors (birth weight, gestational age, infant sex, antenatal corticosteroids, plurality, and hospital of birth), birth weight contributed most to short-term mortality^23^. The birth weights of preterm infants, particularly extremely preterm infants, born to mothers with HDP were considerably lower than those of infants born to mothers without HDP; thus because HDP is frequently accompanied by fetal growth restrictions (Supplementary Fig. 1), the increased crude ORs of adverse outcomes in the HDP group are understandable. However, after adjustment for several covariates, including birth weight, the risks for these adverse outcomes were, in contrast, lower in the HDP group when maternal and fetal characteristics such as gestational age, sex, and birth weight were exactly the same except for the presence of HDP. Therefore, the two-sidedness of HDP infants’ outcomes from different viewpoints should be taken into consideration.

There are several possible explanations for the decreased risk for mortality and severe adverse neurological outcomes in infants born to mothers with HDP if all covariates were identical except for the presence of HDP. First, there may be more infants who develop a poor prognosis in the non-HDP group; second, there may be a higher glucocorticoid level in the HDP group because of the endogenous production of fetal glucocorticoids; third, anti-angiogenic factors such as soluble fms-like tyrosine kinase-1 (sFlt-1) and soluble endoglin (sEng) may inhibit vascular endothelial growth factor (VEGF) signaling, resulting in protection from IVH and fourth, more mothers in the HDP group may have received magnesium sulfate prior to delivery.

It is possible that more infants in the non-HDP group had poor prognostic factors such as severe CAM, placental abruption and fetal distress. CAM is well-known to be associated with poor neurological outcomes in preterm infants^24–26^. However, we found that excluding women with histological CAM from both groups resulted in similar outcomes when all women were included. Additionally, although severe FGR, placental abruption, and non-reassuring fetal status are more likely to be in mothers with HDP in general, the outcomes of the HDP group tended to be better. Unfortunately, our database did not include the causes of preterm delivery (iatrogenic or spontaneous) or information about placental abruption; therefore, we could not exclude these infants. However, the effect of these infants on the overall results may be limited because they were rare.

Another hypothesis is that maternal HDP may have a protective effect on infants’ neurological outcomes. Fetuses in mothers with HDP, especially preeclampsia, are exposed to an adverse intrauterine environment including hypoxia, undernutrition, and various cytokines and anti-angiogenic factors^27–29^. These stressful circumstances may stimulate the endogenous production of fetal glucocorticoids^30,31^, resulting in accelerated fetal lung maturation and protection from severe IVH by the same mechanism as antenatal corticosteroid treatment^32^. This hypothesis is supported by clinical studies showing higher levels of cortisol in umbilical cord blood of pregnancies with preeclampsia or FGR^33,34^. Anti-angiogenic factors, such as sFlt-1 and sEng, are important in the development of HDP^29,35^. According to an in vitro study, these factors may inhibit VEGF signaling and vasculature in the germinal matrix, the usual site of IVH, resulting in protection from IVH^36^. Lastly, magnesium sulfate has a protective effect on severe brain injury in preterm infants^37^, and it is often administered to women with HDP to prevent eclampsia. Unfortunately, the NRNJ database did not include information on the use of magnesium sulfate. For all of these reasons, infants born to mothers with HDP may have a lower incidence of mortality and severe brain injury.

Strengths of this study include the assessment of both short- and medium-term mortality and neurological outcomes in extremely and very preterm infants born to mothers with HDP. In addition, we had a large sample size by accessing a nationwide database. We performed stratification matching to eliminate the effect of confounding variables in the selection of eligible infants. Finally, we performed several subgroups analyses to corroborate the results.

This study did have several limitations. First, the NRNJ database did not include information on certain maternal characteristics such as type and severity of HDP, administration of magnesium sulfate and the causes of preterm delivery (iatrogenic or spontaneous). Second, we were unable to evaluate the influence of NICU management and short-term outcomes on medium-term outcomes. In addition, the rate of follow-up examination at 3 years of age was less than half the initial population. It is possible that more infants with severe impairment were followed up resulting in selection bias. Finally, the aetiology of neurodevelopmental impairment is multifactorial with more causes in preterm than in term infants^38–43^. In addition, preterm birth itself may have an influence on subsequent neurodevelopment^44,45^, and the causes of preterm delivery were unclear in the non-HDP group. Ultimately, it may be difficult to address the differential effect of HDP, prematurity and other factors on subsequent outcomes because all these independent factors occur simultaneously, and all of them may contribute to subsequent neurodevelopmental impairment.

## Conclusion

Extremely and very preterm infants born to mothers with HDP were associated with an increased risk for mortality and adverse neurological outcomes because of lower birth weight; however, they were associated with a lower risk for mortality and adverse neurological outcomes in both the short- and medium-term than those not exposed to HDP, if all covariates were identical except for the presence of HDP. Therefore, the two-sidedness of HDP infants’ outcomes from different viewpoints should be taken into consideration.

## Supplementary information

Supplementary Tables.

Supplementary Figures.

Supplementary Legend.

## Data Availability

The data that support the findings of this study are available from Neonatal Research Network of Japan but restrictions apply to the availability of these data, which were used under license for the current study, and so are not publicly available. Data are however available from the authors upon reasonable request and with permission of Neonatal Research Network of Japan.
