# Supplementary figures and images for: Mortality and neurological outcomes in extremely and very preterm infants born to mothers with hypertensive disorders of pregnancy
Source: Sci Rep. 2021 Jan 18;11:1729. doi: 10.1038/s41598-021-81292-7 (PMC7814115; doi:10.1038/s41598-021-81292-7)

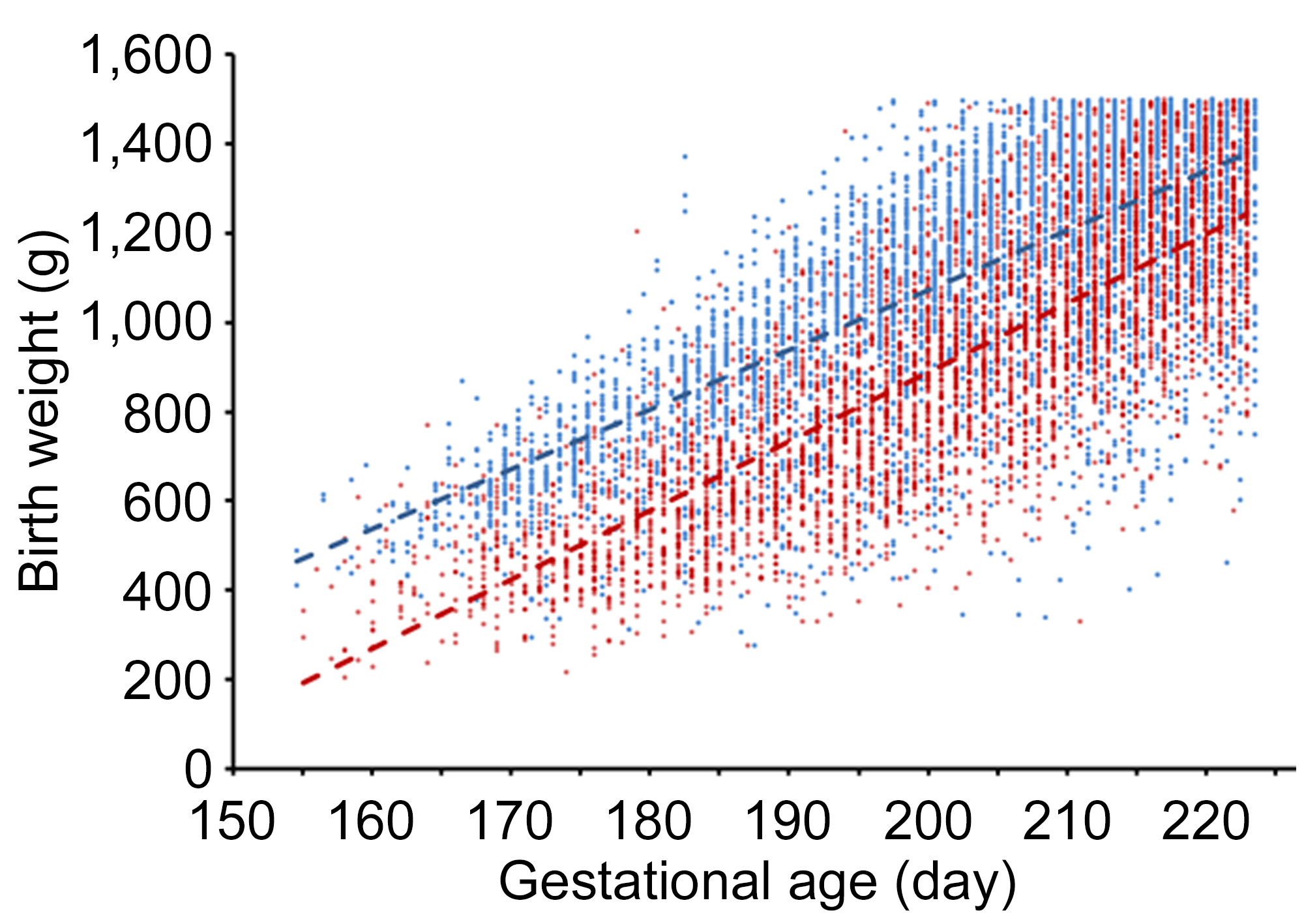

Supplement: Supplementary file 1 — Supplementary Tables. [file 41598_2021_81292_MOESM1_ESM.tif]
