# Supplementary material for: Mortality and neurological outcomes in extremely and very preterm infants born to mothers with hypertensive disorders of pregnancy
Source: Sci Rep. 2021 Jan 18;11:1729. doi: 10.1038/s41598-021-81292-7 (PMC7814115; doi:10.1038/s41598-021-81292-7)
Supplement: Supplementary file 2 — Supplementary Figures. [file 41598_2021_81292_MOESM2_ESM.pdf]

Mortality and neurological outcomes in extremely and very preterm infants born to mothers with hypertensive disorders of pregnancy.

Noriyuki Nakamura[1], Takafumi Ushida[1*], Masahiro Nakatochi[2], Yumiko Kobayashi[3], Yoshinori Moriyama[1,4], Kenji Imai[1], Tomoko Nakano-Kobayashi[1], Masahiro Hayakawa[5], Hiroaki Kajiyama[1], Fumitaka Kikkawa[1], Tomomi Kotani[1,6], for the Neonatal Research Network of Japan

[1]Department of Obstetrics and Gynecology, Nagoya University Graduate School of Medicine, Nagoya, Japan

[2]Division of Public Health Informatics, Department of Integrative Health Science, Nagoya University Graduate School of Medicine, Nagoya, Japan

[3]Data Science Division, Data Coordinating Center, Department of Advanced Medicine, Nagoya University Hospital, Nagoya, Japan

[4]Department of Obstetrics and Gynecology, Fujita Health University Graduate School of Medicine, Toyoake, Japan

[5]Division of Neonatology, Center for Maternal-Neonatal Care, Nagoya University Hospital, Nagoya, Japan

[6]Division of Perinatology, Center for Maternal-Neonatal Care, Nagoya University Hospital,

Nagoya, Japan

Supplementary Table 1. Baseline maternal and neonatal characteristics with and without complete data.

Variables	With complete data (n = 21,659)	Without complete data (n = 7,488)	*p*-value
Maternal characteristics			
Maternal age (years)	31.7 ± 5.5	31.6 ± 5.5	0.02
Primiparous	10,713 (49.5%)	3,797/7,304 (52.0%)	<0.01
Gestational age (weeks)	27.6 ± 2.5	27.8 ± 2.5	<0.01
Caesarean section	16,198 (74.8%)	5,230/7,396 (70.7%)	<0.01
HDP	4,629 (21.4%)	1,417/7,370 (19.2%)	<0.01
DM or GDM	665 (3.1%)	158/7,233 (2.2%)	<0.01
Histological chorioamnionitis	9,224 (42.6%)	660/1,765 (37.4%)	<0.01
Antenatal corticosteroid	12,241 (56.5%)	3,141/7,228 (43.5%)	<0.01
PROM	7,932 (36.6%)	2,463/7,419 (33.2%)	<0.01
Year of delivery			
2003-2008	6,530 (30.1%)	3,710 (49.5%)	<0.01
2009-2015	15,129 (69.9%)	3,778 (50.5%)	
Neonatal characteristics			
Male	11,291 (52.1%)	3,958/7,473 (53.0%)	0.21
Birth weight (g)	954 ± 297	975 ± 300	<0.01
Height (cm)	34.3 ± 4.0	34.8 ± 4.0	<0.01

Data are given as mean ± standard deviation for continuous variables and n (%) for categorical variables. HDP, hypertensive disorders of pregnancy; DM, diabetes mellitus; GDM, gestational diabetes mellitus; PROM, premature rupture of membranes.

Supplementary Table 2. Baseline characteristics before 1:1 matching in the HDP and non-HDP groups

Variables	HDP (n = 4,629)	non-HDP (n = 17,030)	*p*-value	Total (n = 21,659)
Maternal characteristics				
Maternal age (years)	33.9 ± 5.0	31.2 ± 5.4	<0.01	31.7 ± 5.5
Primiparous	2,651 (57.3%)	8,062 (47.3%)	<0.01	10,713 (49.5%)
Gestational age (weeks)	28.8 ± 2.2	27.3 ± 2.4	<0.01	27.6 ± 2.5
Caesarean section	4,450 (96.1%)	11,748 (69.0%)	<0.01	16,198 (74.8%)
DM or GDM	194 (4.2%)	471 (2.8%)	<0.01	665 (3.1%)
Histological chorioamnionitis	649 (14.0%)	8,575 (50.4%)	<0.01	9,224 (42.6%)
Antenatal corticosteroid	2,477 (53.5%)	9,764 (57.3%)	<0.01	12,241 (56.5%)
PROM	189 (4.1%)	7,743 (45.5%)	<0.01	7,932 (36.6%)
Year of delivery			<0.01	
2003-2008	1,191 (25.7%)	5,339 (31.4%)		6,530 (30.1%)
2009-2015	3,438 (74.3%)	11,691 (68.6%)		15,129 (69.9%)
Neonatal characteristics				
Male	2,145 (46.3%)	9,146 (53.7%)	<0.01	11,291 (52.1%)
Birth weight (g)	916 ± 291	964 ± 298	<0.01	954 ± 297
Height (cm)	34.1 ± 4.0	34.3 ± 4.0	<0.01	34.3 ± 4.0
SGA (%)	2,495 (53.9%)	2,010 (11.8%)	<0.01	4,505 (20.8%)

Data are given as mean ± standard deviation for continuous variables and n (%) for categorical variables. HDP, hypertensive disorders of pregnancy; DM, diabetes mellitus; GDM, gestational diabetes mellitus; PROM, premature rupture of membranes; SGA: small for gestational age.

Supplementary Table 3. Mortality and severe neurological outcomes in the HDP and non-HDP groups after excluding cases with histological chorioamnionitis

Variables	HDP	non-HDP	Crude OR (95% CI)	Adjusted OR (95% CI)
Short-term outcomes	(n = 3,941)	(n = 2,541)		
IVH (grade III or IV)	88/3,923 (2.2%)	76/2,527 (3.0%)	0.74 (0.54–1.01)	**0.48 (0.35–0.68)**
Periventricular leukomalacia	93/3.928 (2.4%)	91/2,531 (3.6%)	**0.65 (0.49–0.87)**	**0.61 (0.45–0.84)**
Neonatal seizures	57/3,936 (1.4%)	51/2,540 (2.0%)	0.72 (0.49–1.05)	**0.46 (0.31–0.70)**
In-hospital death	147/3,941 (3.7%)	80/2,540 (3.1%)	1.19 (0.90–1.57)	**0.55 (0.40–0.75)**
Medium-term outcomes	(n = 1,836)	(n = 1,128)		
Cerebral palsy	93/1,608 (5.8%)	69/995 (6.9%)	0.82 (0.60–1.14)	**0.68 (0.48–0.96)**
Developmental quotient of <70	191/1,162 (16.4%)	82/695 (11.8%)	**1.47 (1.11–1.94)**	1.15 (0.84–1.57)
Total death by 3 years of age	157/1,836 (8.6%)	88/1,128 (7.8%)	1.11 (0.84–1.45)	**0.54 (0.39–0.74)**

Data are given as n (%). Bold text indicates a significant association. Multivariate analysis was adjusted by diabetes mellitus or gestational diabetes mellitus, administration of antenatal corticosteroid, mode of delivery, gender, and birth weight. HDP, hypertensive disorders of pregnancy; IVH, intraventricular haemorrhage; OR, odds ratio; CI, confidence interval.

Supplementary Table 4. Mortality and severe neurological outcomes in the HDP and non-HDP groups born prior to 28 weeks of gestation

Variables	HDP	non-HDP	Crude OR (95% CI)	Adjusted OR (95% CI)
Short-term outcomes	(n = 1,486)	(n = 1,486)		
IVH (grade III or IV)	81/1,475 (5.5%)	114/1,478 (7.7%)	**0.70 (0.52–0.93)**	**0.52 (0.36–0.74)**
Periventricular leukomalacia	54/1,478 (3.7%)	67/1,481 (4.5%)	0.80 (0.56–1.15)	0.88 (0.56–1.40)
Neonatal seizures	44/1,480 (3.0%)	61/1,484 (4.1%)	0.72 (0.48–1.06)	**0.42 (0.26–0.68)**
In–hospital death	154/1,486 (10.4%)	105/1,485 (7.1%)	**1.52 (1.17–1.97)**	**0.60 (0.43–0.84)**
Medium–term outcomes	(n = 774)	(n = 743)		
Cerebral palsy	69/580 (11.9%)	54/596 (9.1%)	1.36 (0.93–1.97)	1.08 (0.67–1.75)
Developmental quotient of <70	120/440 (27.3%)	85/434 (19.6%)	**1.54 (1.12–2.11)**	0.92 (0.61–1.39)
Total death by 3 years of age	161/774 (20.8%)	114/743 (15.3%)	**1.45 (1.11–1.89)**	**0.57 (0.41–0.82)**

Data are given as n (%). Bold text indicates a significant association. Multivariate analysis was adjusted by diabetes mellitus or gestational diabetes mellitus, histological chorioamnionitis, administration of antenatal corticosteroid, mode of delivery, gender, and birth weight. HDP, hypertensive disorders of pregnancy; IVH, intraventricular haemorrhage; OR, odds ratio; CI, confidence interval.

Supplementary Table 5. Mortality and severe neurological outcomes in the HDP and non–HDP groups born between 28 and 31 weeks of gestation

Variables	HDP	non–HDP	Crude OR (95% CI)	Adjusted OR (95% CI)
Short–term outcomes	(n = 3,098)	(n = 3,098)		
IVH (grade III or IV)	27/3,090 (0.9%)	43/3,087 (1.4%)	0.62 (0.39–1.01)	**0.58 (0.34–0.99)**
Periventricular leukomalacia	57/3,089 (1.8%)	114/3,087 (3.7%)	**0.49 (0.36–0.68)**	**0.52 (0.36–0.74)**
Neonatal seizures	19/3,097 (0.6%)	37/3,095 (1.2%)	**0.51 (0.29–0.89)**	**0.42 (0.23–0.76)**
In–hospital death	27/3,097 (0.9%)	35/3,097 (1.1%)	0.77 (0.47–1.27)	**0.51 (0.30–0.88)**
Medium–term outcomes	(n = 1,340)	(n = 1,226)		
Cerebral palsy	43/1,265 (3.4%)	66/1,141 (5.8%)	**0.57 (0.39–0.85)**	**0.53 (0.34–0.81)**
Developmental quotient of <70	96/904 (10.6%)	82/830 (9.9%)	1.08 (0.79–1.48)	0.89 (0.63–1.27)
Total death by 3 years of age	31/1,340 (2.3%)	45/1,226 (3.7%)	**0.62 (0.39–0.99)**	**0.49 (0.29–0.80)**

Data are given as n (%). Bold text indicates a significant association. Multivariate analysis was adjusted by diabetes mellitus or gestational diabetes mellitus, histological chorioamnionitis, administration of antenatal corticosteroid, mode of delivery, gender, and birth weight. HDP, hypertensive disorders of pregnancy; IVH, intraventricular haemorrhage; OR, odds ratio; CI, confidence interval.
