# Supplementary material for: Mortality and neurological outcomes in extremely and very preterm infants born to mothers with hypertensive disorders of pregnancy
Source: Sci Rep. 2021 Jan 18;11:1729. doi: 10.1038/s41598-021-81292-7 (PMC7814115; doi:10.1038/s41598-021-81292-7)
Supplement: Supplementary file 3 — Supplementary Legend. [file 41598_2021_81292_MOESM3_ESM.doc]

Supplementary Figure 1. Relationship between gestational age and birth weight in the groups with and without hypertensive disorders of pregnancy (HDP).

Gestational age (day) vs. birth weight (g) with a fitted curve in the HDP group (red; n = 4,584) and the non-HDP group (blue; n = 4,584).
